# Draft genome sequence of a multidrug-resistant *Escherichia coli* O13/O129:H11 isolate from imported beef in Saudi Arabia

**DOI:** 10.1128/mra.00916-24

**Published:** 2025-06-17

**Authors:** Othman I. Aljurayyad, Elaf A. Alshdokhi, Mohammed I. Mujallad, Ashwaq S. Alhamed, Shahad A. Alsalman, Khaloud O. Alzahrani, Saleh I. Al-Akeel, Abdulmohsen L. AlHarbi, Hatim Almutairi, Dalal M. Alkuraythi, Turki M. Dawoud, Sulaiman Alajel, Meshari Ahmed Alhadlaq

**Affiliations:** 1 Saudi Food and Drug Authority (SFDA), Riyadh, Saudi Arabia; 2 Botany and Microbiology Department, Science College, King Saud University (KSU), Riyadh, Saudi Arabia; 3 Public Health Authority (PHA), Riyadh, Saudi Arabia; 4 Biology Department, College of Science, University of Jeddah (UJ), Jeddah, Saudi Arabia; University of Maryland School of Medicine, Baltimore, Maryland, USA

**Keywords:** *Escherichia coli*, O13, O129, O135, food poisoning, meat, imported food

## Abstract

*Escherichia coli*, typically harmless, resides in the gut. However, certain strains are lethal pathogens. Here, we report a draft genome sequence of a multidrug resistance *E. coli* O13/O129 (food source) in Saudi Arabia. O-antigen of *E. coli* O13/O129 is closely related to *Shigella flexneri* (causative agent of shigellosis).

## ANNOUNCEMENT

*Escherichia coli* is a gram-negative bacterium belonging to the Enterobacteriaceae family (1, 2). The serogroups O13 and O129 of *E*. *coli* are closely related with 99% identical and 100% coverage (3). Moreover, O135 strain is also closely related to O13/O129 with 82% coverage (3). The structure of *E. coli* O13/O129 antigens was found to be closely related to the O-antigen structure of *Shigella flexneri* types 1–5 especially type 5a (4). Several *E. coli* strains cause numerous human infections (5, 6).

This announcement is presenting a draft genome sequence of multidrug resistance of *E. coli* O13/O129:H11 (designated identifier name: EC190602) that isolated from imported beef from Sudan during March 2022.

Isolation and identification of EC190602 were conducted according to the total enumeration method of *E. coli* in food products guide, using 3M petrifilm select *E. coli* plates following manufacturer instructions (catalog number 6435). Briefly, a single colony was picked up and cultured in a MacConkey agar plate for 24h at 37°C, and then, a single colony was picked up and identified by MALDI-TOF (Bruker) following the manufacturer protocol (7, 8). An antimicrobial susceptibility test was conducted using the AIM senstiter (auto-inoculator) to measure the minimum inhibitory concentration. Resistance to antibiotics was interpreted using CLSI and NARMS guidelines (9, 10). EC190602 showed resistance to Trimethoprim/Sulfamethoxazole, Tetracycline, Sulfisoxazole, Streptomycin, and Ampicillin. Due to the resistance to more than two antibiotic classes, this pattern is considered a multidrug resistance (9, 10).

For sequencing preparation, EC190602 was cultured in nutrient agar (Oxoid) overnight at 37°C, and then a single colony was picked and transferred into a 1.5 mL tube containing nuclease-free water for the genomic DNA extraction using the QIAcube with QIAamp DNA Mini kit following the manufacturer’s instructions (Qiagen). DNA concentration was measured using Qubit flex machine. A whole-genome sequence library was constructed using the QIAseq FX DNA library preparation kit, and the input concentration was 100 ng of DNA following the manufacturer’s protocol (Qiagen). Library size was 300–350 bp, with paired-end reads of 2 × 150–175 bp. Sequencing was performed using Illumina Novaseq-6000 platform with short reads using two SP flow cells. Data quality was checked prior to analysis, with phred scores measured based on Q30.

Raw reads quality was checked using (version 0.11.9) of the FastQC tool (11). EC190602 data passed quality control with a Q30 average value of 92.32%, total reads 2722145, genome size (bp) 4932653, number of contigs 138, N50 (bp) 194371, GC content 50.74%, genome coverage 129×, and total genes 4,910. Then, the genome was assembled using Unicycler pipeline (version 0.5.0) (12). The RagTag (version 2.1.0) software tool was used for scaffolding and improving genome assemblies (13). TORMES (version 1.3.0) and PGAP were used for data analysis with default parameters and genome annotation, respectively (14, 15).

EC190602 were identified as *E. coli* O13/O129:H11, MLST: ST10, and FimH:fimH27. Details of virulence genes, AMR genes and plasmids are in Table 1.

**TABLE 1:** Summary of genetic determinants of the isolate *E. coli* O13/O129:H11 (EC190602) sequencing: virulence genes, AMR genes and plasmids predicted by Tormes pipeline

General classification	Gene name	Product name and/or virulence mechanism			
Virulence genes	*espX5, espX4, espR1, espL1*	Type III secretion system effectors			
	*fimI, fimC, fimD, fimF, fimG, fimH, fimA, fimE, fimB*	Fimbrial (adhesion) proteins (Type 1 fimbriae)			
	*entD, fepA, fes, entF, fepC, fepG, fepD, entS, fepB, entC, entE, entB, entA*	Enterobactin synthesis and (iron acquisition)			
	*gtrB, gtrA*	Lipopolysaccharide (LPS) biosynthesis		Bactoprenol-linked glucose translocase/flippase	
	*csgG, csgF, csgD, csgB*	Curli formation (Fimbriae-associated proteins)		Curli production assembly and transcriptional regulators	
AMR genes	*mdtP, mdtO, mdtN, emrB, emrA, emrR, mdtA, mdtB, mdtC, mdtE, mdtF, acrF, acrE, acrS, bacA, tolC, mdtM, mdtG, mdtH*	Multidrug resistance efflux pumps			
	*eptA, pmrF, ugd*	Lipid A modification to reduce antibiotic binding			
	*baeS, baeR, cpxA, evgA, evgS, gadW, gadX, marA, H-NS, CRP, kdpE*	Regulatory systems		Regulators promoting or repressing efflux pump expression	
	*ampC, ampC1, ampH, TEM-1*	Beta-lactamase		Beta-lactamase enzymes conferring resistance to beta-lactam antibiotics	
	*tet(A*)	Tetracycline efflux pump			
	*dfrA14*	Dihydrofolate reductase conferring resistance to diaminopyrimidines			
Plasmid	Plasmid name	Coverage (%)	Identity (%)	Contig number	Contig specific number
	Col440I_1	100.0	90.35	JBEHBQ010000080.1	80
	IncY_1	100.0	99.08	JBEHBQ010000066.1	66

The SFDA group suggests a further analysis on *E. coli* O13/O129:H11 to characterize the genome similarity with the shigellosis’s causative agent, *S. flexneri* (16).

## Data Availability

The genome sequence has been deposited in GenBank under accession number JBEHBQ000000000 and assembly number GCA_041294805.1, as well as in the NCBI Sequence Read Archive (SRA) under accession number SRR23652006.
